# A new species of *Occidozyga* (Amphibia, Anura, Dicroglossidae) and new records of *O. lingnanica* from Central Vietnam

**DOI:** 10.3897/zookeys.1278.169620

**Published:** 2026-04-27

**Authors:** Hoa Thi Ninh, Tao Thien Nguyen, Hoang Huy Nguyen, Cuong The Pham, Chung Van Hoang, Vien Hong Thi Nguyen, Dang Trong Do, Truong Quang Nguyen, Nikolai Orlov, Thomas Ziegler

**Affiliations:** 1 Institute of Biology, Vietnam Academy of Science and Technology, 18 Hoang Quoc Viet Road, Hanoi 10072, Vietnam University of Cologne Cologne Germany; 2 Faculty of Resources and Environment, Thai Nguyen University of Sciences, Thai Nguyen University, Phan Dinh Phung Ward, Thai Nguyen 250000, Vietnam Vietnam Academy of Science and Technology Hanoi Vietnam; 3 Phu Yen University, Tuy Hoa Ward, Dak Lak Province, Vietnam Phu Yen University Tuy Hoa Ward Vietnam; 4 Department of Herpetology, Zoological Institute, Russian Academy of Sciences 199034, St. Petersburg, Russia Thai Nguyen University of Sciences, Thai Nguyen University Phan Dinh Phung Ward Vietnam; 5 AG Zoologischer Garten Köln, Riehler Strasse 173, D-50735 Cologne, Germany Zoological Institute, Russian Academy of Sciences St. Petersburg Russia; 6 Institute of Zoology, University of Cologne, Zülpicher Straße 47b, D-50674 Cologne, Germany AG Zoologischer Garten Köln Cologne Germany

**Keywords:** 16S rRNA gene, *Occidozyga nishikawai* sp. nov., Puddle frogs, species complex, taxonomy

## Abstract

A new species of the genus *Occidozyga* is described from Central Vietnam based on collected specimens from Dak Lak, Gia Lai, Khanh Hoa and Quang Ngai provinces. The new species differs from its congeners by small size (SVL of 23.76–27.50 mm in males and 27.86–34.60 mm in females); tongue cordiform, round posteriorly; fingers free of webbing, toes two-thirds webbed; external vocal sac absent in males; males with a nuptial pad on finger I; dorsum relatively smooth with several small tubercles, venter smooth; dorsal surface brown with a dark brown or a yellow stripe. The genetic distance between the new species and other *Occidozyga* taxa is ≥ 5.50% (16S rRNA gene fragment). New records of *Occidozyga lingnanica* are also reported from Gia Lai and Quang Ngai provinces.

## Introduction

The genus *Occidozyga* contains 17 taxa and can be found from West Bengal (India) through Bangladesh, Myanmar, Thailand, Laos, southern China, Vietnam, Malaysia, southwards to Indonesia, and the Philippines (Frost 2026). Flury et al. (2021) evaluated the diversity of this genus using a 5% genetic divergence threshold in the mitochondrial 16S rRNA gene as a criterion for delimiting candidate species. However, recent studies have revealed much lower interspecific distances; for example, *O. myanhessei* (Kohler, Vargas, Than & Thammachoti, 2021) and *O. lingnanica* Lyu & Wang, 2022 exhibit distances as low as 2.2% compared to their closest congeners, and these low values suggest a high potential for discovering additional cryptic species.

Chan et al. (2022) cautioned that relying solely on mtDNA genetic distances may lead to an overestimation of species diversity due to factors like gene flow (Flury et al. 2021; Chan et al. 2022; Lyu et al. 2022). Therefore, a multi-locus approach and integrative taxonomic evidence are essential for accurately delimiting new taxa within the *Occidozyga* complex.

Since 2021, five new species have been described based on integrative taxonomy combining molecular and morphological analyses. Specifically, *Occidozyga myanhessei*, *O. swanbornorum* (Trageser, Al-Razi, Maria, Nobel, Asaduzzaman & Rahman, 2021), and *O. lingnanica* were described from populations previously assigned to *O. martensii* (Peters, 1867) (Köhler et al. 2021; Trageser et al. 2021; Lyu and Wang 2022). Meanwhile, *O. berbeza* Matsui, Nishikawa, Eto, Hamidy, Hossman & Fukuyama, 2021 and *O. shiwandashanensis* Chen, Peng, Liu, Huang, Liao & Mo, 2022 were recently described as distinct lineages within the genus (Matsui et al. 2021; Chen et al. 2022). These findings demonstrate a high potential for discovering cryptic diversity within *Occidozyga*.

In Vietnam, *Occidozyga* species are widely distributed from northern (e.g., Tuyen Quang and Dien Bien provinces) to southern (e.g., Dak Lak and Vietnam Dong Nai provinces) with three species reported so far. While *O. lima* (Gravenhorst, 1829) and *O. martensii* have wide distributions throughout the country, *O. shiwandashanensis* is only reported from Bac Ninh Province (Nguyen et al. 2009; Tran et al. 2023). During our fieldwork in Central Vietnam, we collected two series of small Puddle Frogs (*Occidozyga*) previously assigned to *O. martensii*. Closer morphological examination showed that while one new collected series was *O. lingnanica*, the second lineage could be clearly distinguished from other *Occidozyga* species by a combination of morphological features. Phylogenetic analyses revealed that this taxon clustered within the *O. martensii* group with strong support, but was distinctly separated from congeners. Due to morphological and molecular differences between the newly collected specimens and all known species in the genus, we herein describe the recently discovered *Occidozyga* population from central Vietnam as a new species.

## Material and methods

### Sampling

Field surveys were conducted in Hoa Thinh Commune, Dak Lak Province and Van Ninh Commune, Khanh Hoa Province in April 2022 by C. T. Pham, T. Q. Nguyen, T. Q. Phan, H. Q. Nguyen, D. T. Do; in Kon Plong Commune, Quang Ngai Province and Kon Chu Rang Nature Reserve and Kon Ka Kinh National Park, Gia Lai Province in May 2023 by H. T. Ninh, H. Q. Nguyen, N. T. Nguyen, C. V. Hoang, N. Orlov.

Specimens were collected between 19:00 and 23:30 h. After taking photographs, specimens were anaesthetized and euthanized in a closed vessel with a piece of cotton wool containing ethyl acetate (Simmons 2002), fixed in 80% ethanol for 4–6 hours, and then transferred to 70% ethanol for permanent storage. Tissue samples were preserved separately in 95% ethanol. Preserved specimens were deposited at the Institute of Biology (**IB**), Hanoi, Vietnam.

### Molecular data and phylogenetic analyses

We used the protocols of Kuraishi et al. (2013), modified by Nguyen et al. (2015a), for DNA extraction, amplification, and sequencing. Fragments of 16S rRNA (mitochondrial DNA) were amplified using the primers following Kuraishi et al. (2013). Three sequences of *Meristogenys jerboa* (Günther, 1872), *M. kinabaluensis* (Inger, 1966) and *M. stenocephalus* Shimada, Matsui, Yambun & Sudin, 2011 were selected as outgroups and the new sequences in this study were deposited in GenBank (see Table 1).

**Table 1.** List of samples of *Occidozyga* and other species used for phylogenetic analysis in this study.

No	Species	Voucher	Locality	GenBank No	References
1	*Occidozyga nishikawai* sp. nov.	IB A.6298	Hoa Thinh, Dak Lak, Vietnam	PP723119	This study
2	*Occidozyga nishikawai* sp. nov.	IB A.6289	Hoa Thinh, Dak Lak, Vietnam	PP723120	This study
3	*Occidozyga nishikawai* sp. nov.	IB A.6292	Kon Plong, Quang Ngai, Vietnam	PP723121	This study
4	*Occidozyga nishikawai* sp. nov.	IB A.6294	Kon Chu Rang Nature Reserve, Gia Lai, Vietnam	PP723122	This study
5	*Occidozyga nishikawai* sp. nov.	IB A.6287	Kon Plong, Quang Ngai, Vietnam	PP723123	This study
6	*Occidozyga nishikawai* sp. nov.	IB A.6295	Kon Plong, Quang Ngai, Vietnam	PP723124	This study
7	*Occidozyga nishikawai* sp. nov.	IB A.6288	Kon Plong, Quang Ngai, Vietnam	PP723125	This study
8	*Occidozyga nishikawai* sp. nov.	IB A.6296	Kon Plong, Quang Ngai, Vietnam	PP723126	This study
9	*Occidozyga nishikawai* sp. nov.	IB A.6293	Kon Plong, Quang Ngai, Vietnam	PP723127	This study
10	*O. baluensis*	SH0167	Danum Valley Conservation Area, Sabah, Malaysia	MW007191	Flury et al. 2021
11	*O. baluensis*	SH0310	Tawau Hills National Park, Sabah, Malaysia	MW007203	Flury et al. 2021
12	*O. berbeza*	KUHE:53037	Matang, Sarawak, Malaysia	LC593608	Matsui et al. 2021
13	*O. berbeza*	KUHE:57073	Matang, Sarawak, Malaysia	LC593610	Matsui et al. 2021
14	*O. cf. lima*	Alive individual	Java, Indonesia	AB530619	Hasan et al. 2014
15	*O. cf. martensii*	Alive individual	Ranong, Thailand	AB530610	Hasan et al. 2014
16	*O. diminutiva*	KU 321225	Pasonanca, Zamboanga, Mindanao, Philippines	MT820199	Chan et al. 2021
17	*O. diminutiva*	KU 321226	Pasonanca, Zamboanga, Mindanao, Philippines	MT820200	Chan et al. 2021
18	*O. laevis*	PNM 7446	Lao, Quezon, Philippines	AY313684	Evans et al. 2003
19	*O. laevis*	RMB 16437	Municipality of Gingoog City, Misamis Oriental Province, Philippines	MW007239	Flury et al. 2021
20	*O. laevis*	HEP-02632	Tawau Hills National Park, Sabah, Malaysia	MW007244	Flury et al. 2021
21	*O. laevis*	SH0277	Tawau Hills National Park, Sabah, Malaysia	MW007257	Flury et al. 2021
22	*O. lima*	CAS 213254	Hlaw Ga Park, Yangon, Myanmar	DQ283224	Frost et al. 2006
23	*O. lingnanica*	IB A.6489	Kon Plong, Quang Ngai, Vietnam	PP723128	This study
24	*O. lingnanica*	IB A.6490	Kon Ka Kinh National Park, Vietnam	PP723129	This study
25	*O. lingnanica*	266003	Phu Luang Wildlife sanctuary, Phu Rua, Loei, Thailand	MW007306	Flury et al. 2021
26	*O. lingnanica*	SYS a002967	Jinghong, Yunnan, China	ON615088	Lyu et al. 2022
27	*O. magnapustulosa*	A1886-12	Doi Mussoe, Tak, Thailand	MW007299	Flury et al. 2021
28	*O. magnapustulosa*	A2065_14	Kalasin, Thailand	MW007300	Flury et al. 2021
29	*O. martensii*	268332	Huay Yang National Park, Prachuap Kiri Khan Prov, Thailand	MW007313	Flury et al. 2021
30	*O. myanhessei*	KU 321227	Zamboanga, Mindanao, Philippines	MW144500	Chan et al. 2021
31	*O. myanhessei*	SMF 103797	East Yangon University, Yangon, Myanmar	MW144501	Chan et al. 2021
32	*O. myanhessei*	CAS 220517	Yangon Division, Mainland SE Asia, Myanmar	MW144502	Chan et al. 2021
33	*O. myanhessei*	CAS 220545	Yangon Division, Myanmar	MW144503	Chan et al. 2021
34	*O.* “*rhacoda*”	NMBE 1069835	Usun Apau, Sarawak, Malaysia	MW007274	Flury et al. 2021
35	*O.* “*rhacoda*”	SH13-192	Payeh Maga, logging road to High Camp, Sarawak, Malaysia	MW007280	Flury et al. 2021
36	*O.* “*rhacoda*”	NMBE 1066057	Payeh Maga, Sarawak, Malaysia	MW007281	Flury et al. 2021
37	*O. shiwandashanensis*	NNU 202103284	Mt Shiwandashan, Guangxi, China	MZ747455	Chen et al. 2022
38	*O. shiwandashanensis*	NNU 202103320	Mt Shiwandashan, Guangxi, China	MZ747457	Chen et al. 2022
39	*O. sumatrana*	MZB Amph 16392	Java, Indonesia	LC593611	Matsui et al. 2021
40	*O. sumatrana*	AJT0251	Bukit Barisan Selatan National Park, Sumatra, Indonesia	MW007271	Flury et al. 2021
41	*O. sumatrana*	AJT0249	Bukit Barisan Selatan National Park, Sumatra, Indonesia	MW007272	Flury et al. 2021
42	*O. swanbornorum*	HAR-2019	Chattogram, Bangladesh	MN705433	Trageser et al. 2021
43	*O. swanbornorum*	HAR-2019	Chattogram, Bangladesh	MN705434	Trageser et al. 2021
44	*O. swanbornorum*	HAR-2019	Chattogram, Bangladesh	MN705436	Trageser et al. 2021
45	*M. stenocephalus*	UMS: BORNEENSIS 8684	Sabah, Crocker Range National Park, Kimanis, Malaysia	AB526612	Shimada et al. 2011
46	*M. kinabaluensis*	MBE1064113	Matang, Sarawak, Malaysia	MW219583	Arifin et al. 2021
47	*M. jerboa*	KUHE:12028	Matang, Sarawak, Malaysia	AB526608	Shimada et al. 2011

CHROMAS PRO software (Technelysium Pty Ltd., Tewantin, Australia) was used to edit the sequences, which were aligned using MAFFT version 7 (Katoh and Standley 2013) with default settings. We then checked the initial alignments by eye and adjusted them slightly. We constructed phylogenetic trees using Maximum Likelihood (ML) and Bayesian Inference (BI) approaches. Maximum likelihood trees were constructed using IQ-TREE (Nguyen et al. 2015b) with maximum likelihood bootstrap support (MLBS) evaluated by ultrafast bootstrap approximation with 1000 replicates (Hoang et al. 2018). Prior to Bayesian analyses, we chose the optimum substitution models for entire sequences by using ModelFinder implemented in IQ-TREE based on the Bayesian information criterion (BIC) (Kalyaanamoorthy et al. 2017). According to ModelFinder, the best-fit model for ML analysis was TIM2+F+I+G4. Because the TIM2 model and F parameter are not implemented in MrBayes, we selected the next best-fit model for our Bayesian-inference (BI) analysis, which was the general time reversible model (GTR; Tavaré 1986) with a proportion of invariable sites and a gamma shape parameter. The BI phylogenetic construction was done in MrBayes version 3.2.7a (Ronquist et al. 2012) with two independent runs of four Markov Chains for 10,000,000 generations. A tree was sampled every 100 generations and a consensus topology was calculated for 70,000 trees after discarding the first 30,001 trees (burn-in 1,000,000). We checked parameter estimates and convergence using TRACER version 1.5 (Rambaut and Drummond 2009). We regarded tree nodes in the ML tree with bootstrap values of 95% or greater as sufficiently resolved (Hoang et al. 2018), and nodes with a BPP of 0.95 or greater as significant in the BI analysis (Leaché and Reeder 2002).

### Morphological characters

Measurements were taken with digital calipers to the nearest 0.1 mm. Abbreviations are as follows for a total of 24 measurements: **SVL**: Snout-vent length, **HW**: Head width (across articulation of jaws), **HL**: Head length (from articulation of mandible to anterior tip of snout), **MN**: Distance from jaw angle to nostril, **MFE**: Distance from articulation of mandible to front of the eye, **MBE**: Distance from articulation of mandible to back of the eye, **SL**: Snout length (from anterior corner of eye to tip of snout), **EL**: Eye diameter, **UEW**: Maximum width of upper eyelid, **IND**: Internarial distance, **IOD**: Interorbital distance (minimal distance between orbits), **DAE**: Distance between anterior corners of eyes, **DPE**: Distance between posterior corners of eyes, **S-NL**: Distance from nostril to tip of snout, **N-EL**: Distance from anterior corner of eye to nostril, **FLL**: Upper arm length (from axilla to elbow), **HAL**: Hand length (from elbow to tip of third finger), **F1-4**: Length of fingers I-IV (from base of finger to tip), **THIGH**: Thigh length (from vent to knee), **TL**: Tibia length (from knee to tarsus), **FoL**: Foot length (from tibiotarsal joint to tip of fourth toe) (Matsui et al. 2021). Terminology for describing the webbing formula followed (Glaw and Vences 2007). Sex was determined by the presence of nuptial pads and via gonadal inspection.

## Results

### Phylogenetic analyses

**Figure 1.**
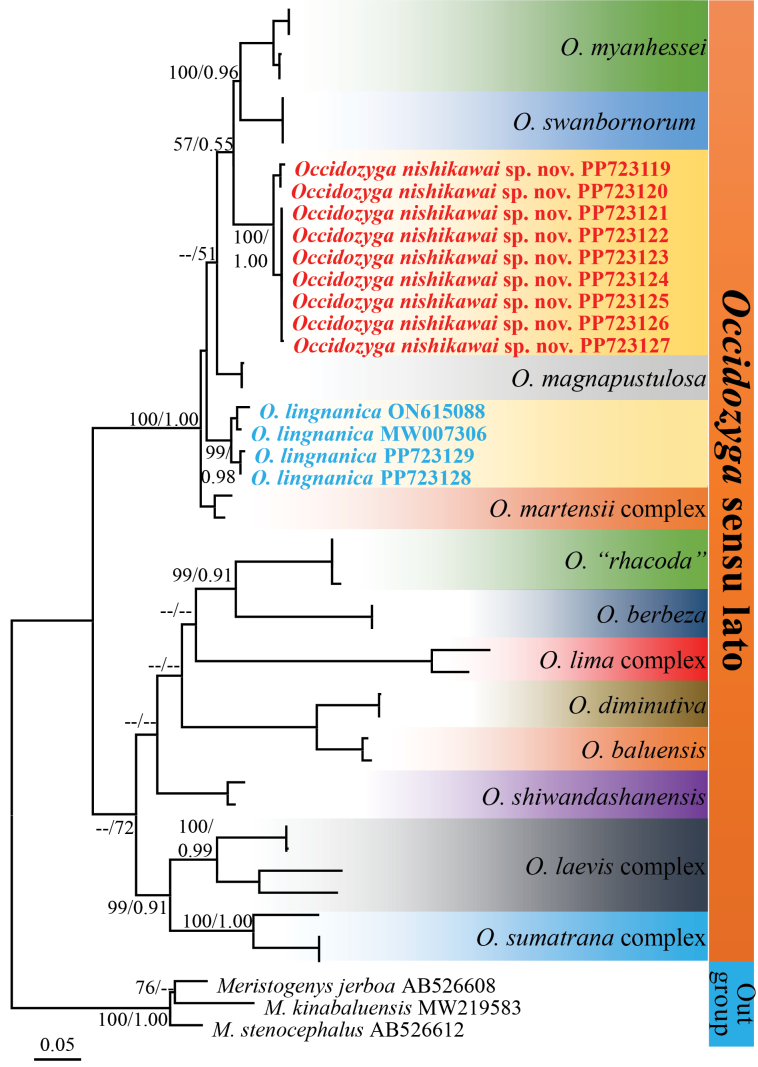
BI tree based on 800 bp sequence of mitochondrial 16S rRNA gene of *Occidozyga* and outgroup species. Numbers above and below branches are Bayesian posterior probabilities and ultrafast-bootstrap (BPP/ML). For GenBank accession numbers, refer to Table 1.

Aligned combined sequences yielded a total of 800 characters, of which 338 were variable and 307 were parsimony-informative within the in-group. Nucleotide frequencies were A = 34.1%, T = 24.4%, C = 23.2%, and G = 18.3% (data for ingroup only). Phylogenetic analyses employing ML and BI methods yielded similar topologies, and only the BI tree is presented in Fig. 1.

The interspecific uncorrected genetic p-distances at the fragment of 16S rRNA gene among *Occidozyga* species ranged from 4.98% (between *O. lingnanica* and *O. cf. martensii*) to 21.14% (between *O. berbeza* and *O. martensii*). In addition, the genetic distance within species was up to 2.28% (*O. myanhessei*). Therein the pairwise distance from the first new collection (KP.2023.8, KKK.2023.80) to the original population of *O. lingnanica* ranged from 0.21–2.78%, confirming these specimens were *O. lingnanica*.

The second new collection of *Occidozyga* sp. was placed in the clade containing *O. myanhessei*, *O. swanbornorum*, *O. magnapustulosa*, *O. lingnanica* and *O. martensii* species complex with strong support (MLBS = 100%, BPP = 1.00). The genetic distance between the other congeners varied from approximately 5.50% (compared with *O. cf. martensii*) to 21.57% (compared with *O.* “*rhacoda*”), and the genetic distance within the candidate species ranged from 0.0–2.12% (Table 2).

**Table 2.** Uncorrected pairwise distances (p-distance) among *Occidozyga* species analyzed.

	Species	1	2	3	4	5	6	7	8	9	10	11	12	13	14	15	16
1	*Occidozyga nishikawai* sp. nov.	0.0–2.12															
2	*O. swanbornorum*	5.58–6.64	0.0–0.0														
3	*O. myanhessei*	5.54–8.76	5.82–6.19	0.0–2.28													
4	*O. magnapustulosa*	5.56–6.9	5.63–5.81	6.68–7.22	0.12–0.13												
5	*O. lingnanica*	5.55–8.82	6.06–6.82	7.59–8.38	5.4–5.95	0.21–2.78											
6	*O. martensii*	5.5–7.99	5.76–6.16	7.3–7.83	5.24–5.92	4.98–6.04	0.53–2.43										
7	*O.* “*rhacoda*”	13.18–21.57	11.97–18.47	17.66–20.75	16.36–20.57	15.32–20.72	16.93–21.07	11.75–14.97	0.0–0.76								
9	*O. lima*	15.91–20.03	16.24–17.39	17.18–19.33	16.6–18.16	16.47–18.52	17.37–18.42	17.67–18.8	17.11–21.14	0.93–7.05							
10	*O. diminutiva*	19.19–20.79	17.13–17.46	19.33–20.64	18.67–18.99	18.51–19.35	19.07–19.64	16.84–17.21	15.22–17.12	19.97–20.89	0.13–0.13						
11	*O. baluensis*	17.17–19.97	15.99–16.51	18.21–19.74	18.07–18.55	17.41–18.29	18.19–19.02	16.33–16.71	16.67–18.31	18.72–19.1	8.27–8.58	0.37–1.27					
12	*O. shiwandashanensis*	14.39–16.55	13.63–13.84	15.05–15.82	14.65–15.31	14.5–15.68	13.5–14.68	12.79–13.68	13.28–16.59	15.94–17.69	15.01–15.95	14.19–14.58	0.49–2.16				
13	*O. sumatrana*	15.98–18.41	14.65–15.68	16.65–17.46	16.01–16.33	15.23–16.67	15.5–16.58	16.26–17.05	16.19–19.97	19.17–20.54	16.15–16.51	15.9–16.56	14.01–14.47	0.0–9.31			
14	*O. laevis*	14.74–18.35	14.73–16.39	14.43–18.04	13.41–17.18	14.41–17.65	14.43–17.12	16.22–17.47	14.92–19.4	18.1–20.26	16.78–18.02	17.8–18.75	13.32–14.92	14.14–15.56	0.13–11.86		

Furthermore, *Occidozyga* sp. is also clearly separated morphologically from all other species of *Occidozyga*. Thus, we conclude that the new lineage from Central Vietnam is a distinct species and describe it below.

### Taxonomic account

#### Occidozyga nishikawai sp. nov.

Taxon classificationAnimaliaAnuraDicroglossidae

C1EC33F5-DD0E-52F9-B8DF-9F9E6FB227EC

https://zoobank.org/CBA96C40-C108-48F8-9C0E-36273F7AFBF7

##### Material examined.

***Holotype*: Vietnam** • ♂; Kon Plong Commune, Quang Ngai Province, Central Vietnam; 1.180 m a.s.l.; April 2023; H. T. Ninh and N. Orlov leg.; IB A.6287 (field number KP.2023.319). ***Paratypes*: Vietnam** • ♂; same locality as for holotype; same collection date; same collectors; IB A.6288 (field number KP.2023.318); • 2♂; Hoa Thinh Commune, Dak Lak Province; C. T. Pham and T. Q. Nguyen leg.; April 2022; IB A.6289 (field number PY.2022.37); IB A.6290 (field number PY.2022.48); • ♂; Van Ninh Commune, Khanh Hoa Province; C. T. Pham and T. Q. Nguyen leg.; April 2022; IB A.6291 (field number KH.2022.22). ♀; Kon Chu Rang National Reserve, Gia Lai Province; H. T. Ninh and O. Nikolai leg.; April 2023; IB A.6494 (field number KCR.2023.159); • 2♀; same locality as for holotype; same collection date; same collectors; IB A.6295 (field number KP.2023.6), IB A.6296 (field number KP.2023.187); • 5♀; Hoa Thinh Commune, Dak Lak Province; C. T. Pham and T. Q. Nguyen leg.; April 2022; IB A.6297 (field number PY.2022.36), IB A.6298 (field number PY.2022.38); IB A.6486 (field number PY.2022.66), IB A.6487 (field number PY.2022.90), IB A.6488 (field number PY.2022.119). 2 juveniles; same locality as for holotype; same collection date; same collectors; IB A.6292 (field number KP.2023.86), IB A.6293 (field number KP.2023.28).

##### Diagnosis.

**Figure 2.**
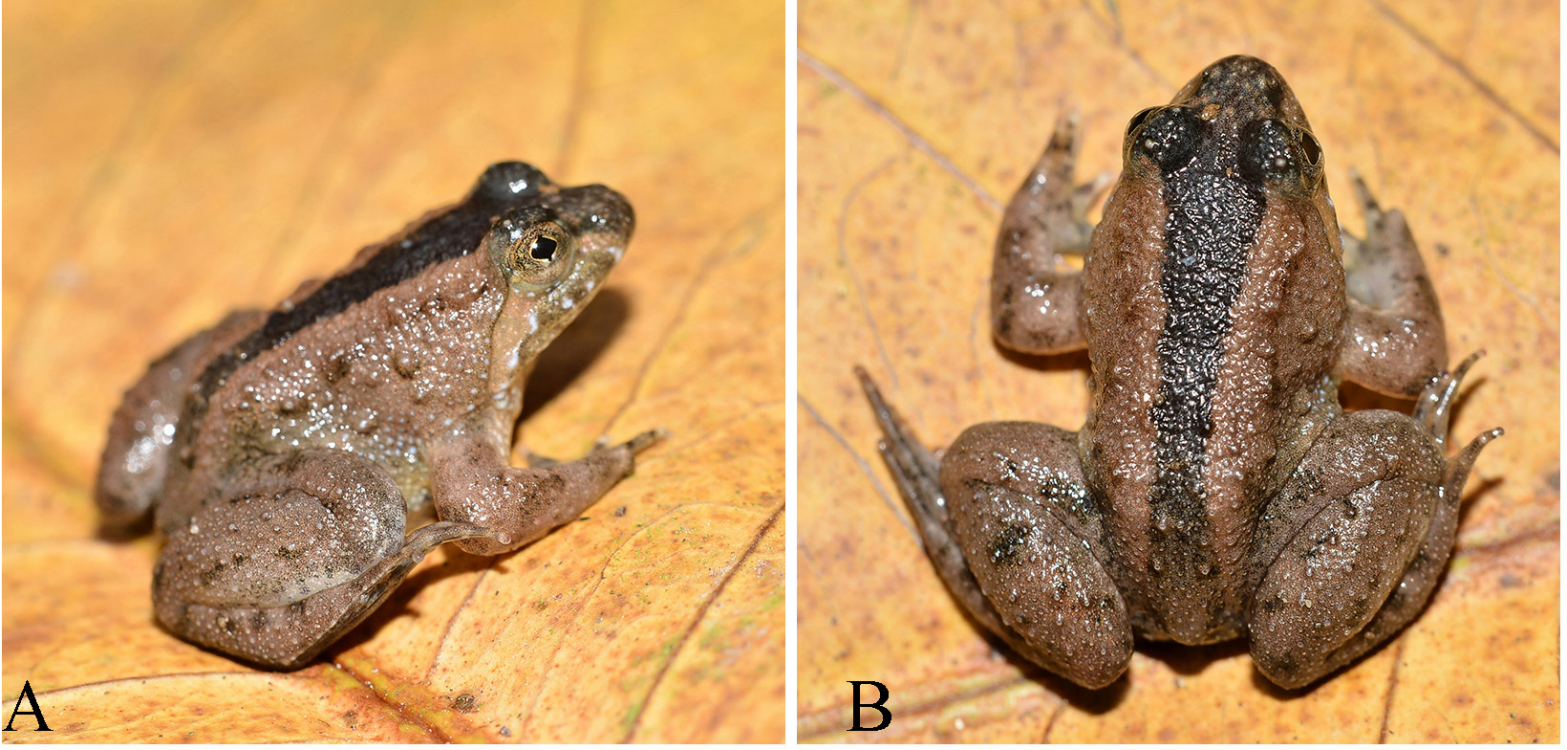
Lateral view (**A**), and dorsal view (**B**) of the adult male holotype (IB A.6287) of *Occidozyga nishikawai* sp. nov. from Kon Plong Commune, Quang Ngai Province, Central Vietnam in life.

Morphologically, *Occidozyga nishikawai* sp. nov. showed the following diagnostic characteristics separating it from other species in the genus *Occidozyga*: (1) size small (SVL of 23.76–27.50 mm in males and 27.86–34.60 mm in females); (2) tongue cordiform, round posteriorly; (3) fingers free of webbing, toes 2/3 webbed; external vocal sac absent in males; males with a nuptial pad on finger I; (4) dorsum relatively smooth with several small tubercles, venter smooth; dorsal surface brown with a dark brown or a yellow stripe (Fig. 2).

##### Description of the holotype.

Size small (SVL 26.96 mm); head longer than wide (HW/HL 0.97); snout round in dorsal and lateral views, projecting slightly over lower jaw; snout short (SL/HL 0.30); canthus rostralis round; loreal region oblique, slightly concave; nostril round, positioned laterally and located at the middle between tip of snout and eye (S-NL/N-EL 0.99); eye orientation laterally; interorbital space distinctly narrower than internarial distance (IOD/IND 0.75); tympanum round, discernible but unclear; vomerine teeth absent; absence of cusp on mandible; tongue cordiform, round posteriorly; external vocal sac absent (Table 3).

**Table 3.** Measurements (mm) of *Occidozyga nishikawai* sp. nov. and *O. lingnanica* in this study. The asterisk (*) indicates the holotype.

Scientific name	*Occidozyga nishikawai* sp. nov.															*Occidozyga lingnanica*	
IB Voucher	IB A.6287*	IB A.6288	IB A.6289	IB A.6290	IB A.6291	IB A.6294	IB A.6295	IB A.6296	IB A.6297	IB A.6298	IB A.6486	IB A.6487	IB A.6488	IB A.6292	IB A.6293	IB A.6489	IB A.6490
Sex	M	M	M	M	M	F	F	F	F	F	F	F	F	J	J	M	F
SVL	26.96	23.76	27.50	26.20	24.00	28.04	27.86	30.99	34.60	33.60	32.10	31.40	33.40	19.50	20.30	22.31	25.93
HW	9.34	8.60	8.60	9.10	7.70	10.50	9.25	11.25	11.40	10.50	10.40	10.10	10.40	6.58	6.92	7.53	9.19
HL	9.65	8.83	9.40	9.30	8.60	11.62	9.91	12.12	12.00	11.70	11.60	11.50	11.60	7.38	7.53	8.13	9.28
MN	8.55	7.85	8.70	8.30	7.00	9.42	8.26	9.72	10.20	9.60	10.20	9.40	9.90	6.57	6.50	7.15	8.09
MFE	6.96	6.21	6.71	6.85	5.90	7.30	6.69	8.25	8.89	8.02	8.75	7.97	7.60	5.46	5.23	5.74	6.56
MBE	4.49	3.61	4.74	4.23	3.55	4.52	4.02	5.06	5.04	5.26	6.03	4.89	4.60	3.05	3.21	3.45	4.04
SL	2.90	2.74	3.50	3.20	3.10	3.82	3.04	3.94	4.10	4.10	4.00	4.00	4.10	2.37	2.72	2.59	3.55
EL	2.76	2.71	3.30	3.00	2.90	3.16	3.21	3.44	4.20	4.10	4.00	3.80	4.00	2.53	2.23	2.50	2.83
UEW	1.71	1.89	1.70	1.60	1.50	1.99	2.01	2.33	2.60	2.50	2.20	2.00	2.40	1.85	1.68	1.88	1.64
IND	2.63	2.40	2.60	2.80	2.40	2.52	2.53	2.74	3.40	3.20	2.80	3.10	3.10	1.93	2.34	2.14	2.56
IOD	1.98	1.83	2.00	1.80	1.70	2.02	2.00	2.13	2.60	2.40	2.21	2.40	2.40	2.01	2.10	2.01	2.46
DAE	3.72	3.61	4.20	4.61	3.56	4.48	4.14	5.46	4.97	5.28	4.92	5.27	5.44	3.27	3.70	3.81	4.27
DPE	6.03	5.73	6.38	6.30	5.64	6.47	6.22	7.93	8.49	8.99	7.72	8.53	7.73	5.14	5.23	5.35	5.51
S-NL	1.83	1.85	1.61	1.52	1.40	2.05	1.93	1.93	2.10	2.12	1.76	2.14	2.10	1.23	1.16	1.97	1.99
N-EL	1.85	1.81	1.59	1.46	1.30	2.08	1.92	1.89	2.11	2.09	1.81	2.09	2.09	1.13	1.12	2.01	2.05
FLL	5.42	4.26	4.60	5.20	4.40	4.64	5.24	5.25	5.60	6.10	5.80	5.80	5.40	3.37	3.93	3.50	4.63
HAL	10.11	9.58	11.30	10.70	9.20	12.01	11.41	13.63	12.70	13.10	12.90	13.60	12.80	8.33	8.78	9.05	9.96
F1	2.75	2.43	2.86	2.41	2.42	2.95	2.98	3.01	3.02	2.85	2.76	2.78	3.01	2.57	2.66	2.39	2.34
F2	2.20	2.12	2.63	2.06	2.04	2.63	2.62	2.65	2.52	2.46	2.39	2.35	2.54	1.95	2.30	2.36	2.32
F3	3.57	2.79	3.90	3.20	2.80	4.16	4.24	3.78	4.00	3.90	3.70	3.90	3.50	2.48	3.34	3.05	3.01
F4	2.81	2.51	2.94	2.46	2.51	3.05	3.01	3.21	3.19	2.89	2.81	2.86	3.12	2.58	2.57	2.55	2.58
THIGH	11.03	11.09	13.10	13.10	11.70	13.00	13.19	15.63	15.50	16.30	15.00	16.10	15.90	8.50	10.10	10.15	12.68
TL	10.73	10.66	12.80	12.40	11.10	12.80	12.93	15.54	15.00	15.00	14.80	15.30	15.20	8.32	9.81	9.03	12.57
FoL	17.74	15.43	14.40	14.20	13.20	20.36	19.15	21.65	15.20	15.30	15.40	16.80	15.00	13.14	14.18	13.48	18.58
HW/HL	0.97	0.97	0.91	0.98	0.90	0.90	0.93	0.93	0.95	0.90	0.90	0.88	0.90			0.93	0.99
IOD/IND	0.75	0.76	0.77	0.64	0.71	0.80	0.79	0.78	0.76	0.75	0.79	0.77	0.77	1.04	0.90	1.06	1.04
SL/HL	0.30	0.31	0.37	0.34	0.36	0.33	0.31	0.33	0.34	0.35	0.34	0.35	0.35	0.32	0.36	0.32	0.38
S-NL/N-EL	0.99	1.02	1.01	1.04	1.08	0.99	1.01	1.02	1.00	1.01	0.97	1.02	1.00	1.09	1.04	0.98	0.97
HAL/SVL	0.38	0.40	0.41	0.41	0.38	0.43	0.41	0.44	0.37	0.39	0.40	0.43	0.38	0.43	0.43	0.41	0.38
TbL/FeL	0.97	0.96	0.98	0.95	0.95	0.98	0.98	0.99	0.97	0.92	0.99	0.95	0.96	0.98	0.97	0.89	0.99
SNL/ED	1.05	1.01	1.06	1.07	1.07	1.21	0.95	1.15	0.98	1.00	1.00	1.05	1.03	0.94	1.22	1.04	1.25

Forelimbs short and robust (HAL/SVL 0.38), fingers thin and long, relative finger lengths II<I<IV<III; tips of fingers pointed, not dilated into disks; fingers free of webbing; subarticular tubercles prominent and round, formula 1,1,2,2 inner and outer palmar tubercles prominent and round; finger I with nuptial pad.

Hind limbs robust, femur shorter than tibia length (THIGH/FL 0.97), heels not meeting when hind limbs flexed at right angles to the axis of the body; inner metatarsal tubercle reaching at the posterior margin of supratympanic rim when the hind limb is stretched along the side of the body; toes long and thin, relative lengths I<II<V<III<IV; tips of toes pointed; webbing formula between toes I ½ – ½ II ½ – 1½ III ½ – 1 IV1 ¾ – ½ V; indistinct lateral fringes on lateral edges of toes I and V; subarticular tubercles round, prominent, formula 1,1,2,3,2; inner metatarsal tubercle elongated; outer metatarsal tubercle absent; tarsal fold absent.

Skin on dorsal surface of the body is relatively smooth, with several round tubercles creating two weak dorsoventral folds; the dorsal surface of thighs is smoother than that of the tibiae with some small tubercles; supratympanic fold distinct, slightly curved and extending from the posterior corner of the eye to shoulder; chin, throat, and chest smooth, belly slightly smooth; ventral side of fore and hindlimbs smooth.

##### Coloration of holotype in life.

Dorsal surface brown with a dark brown stripe, which is paler in head and posterior of the back, along the spine; dorsal limbs with some dark brown spots without transverse bars; a narrow transverse bar between orbits; supratympanic fold brown. Loreal region, flank light brown. Pupil is bordered with bronze margin; iris black. Throat dark with cream mottling; the skin of chest and belly creamy with white speckles; the ventral surface of thighs brownish pink with dark spots, ventral surface of feet dark grey with bronze speckles (Fig. 2).

##### Coloration of holotype in preservative.

**Figure 3.**
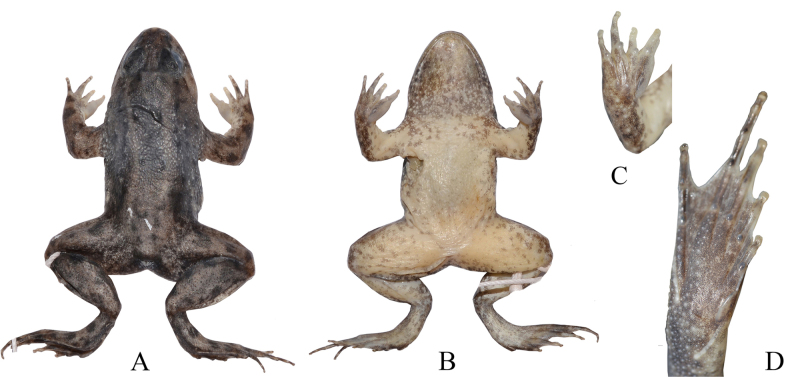
Dorsal view (**A**), ventral view (**B**), ventral side of right hand (**C**), ventral side of right foot (**D**) of the holotype of *Occidozyga nishikawai* sp. nov. (IB A.6287) in preservative.

The dorsum is dark grey; the mid-dorsal stripe is unclear; the nuptial pad is light grey, slightly transparent; the ventral surface is light brown; mottling on the throat grey white (Fig. 3).

##### Variation.

**Figure 4.**
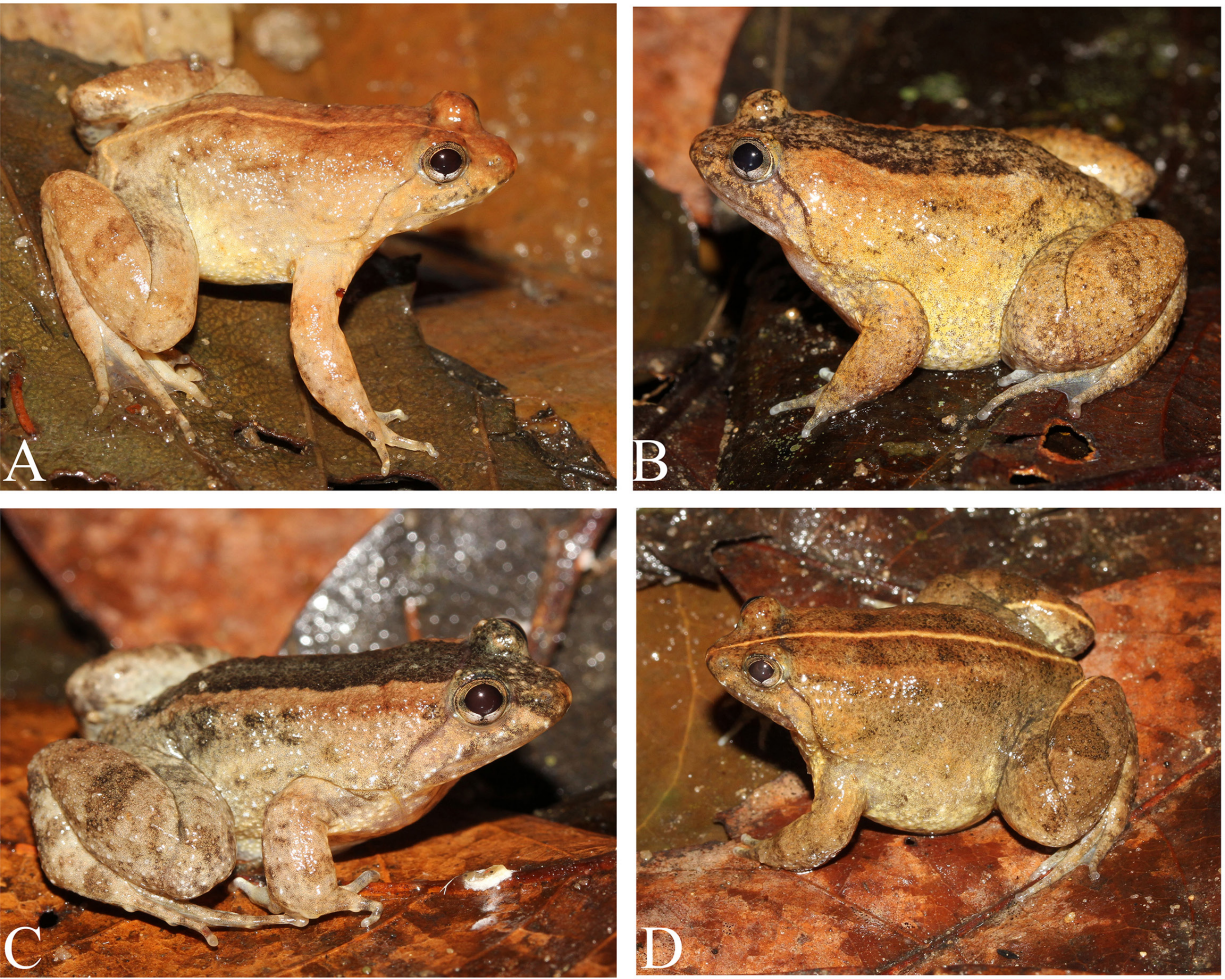
Coloration variation of the paratypes of *Occidozyga nishikawai* sp. nov. in life. **A**. IB A.6290; **B**. IB A.6297; **C**. IB A.6289; **D**. IB A.6291.

**Figure 5.**
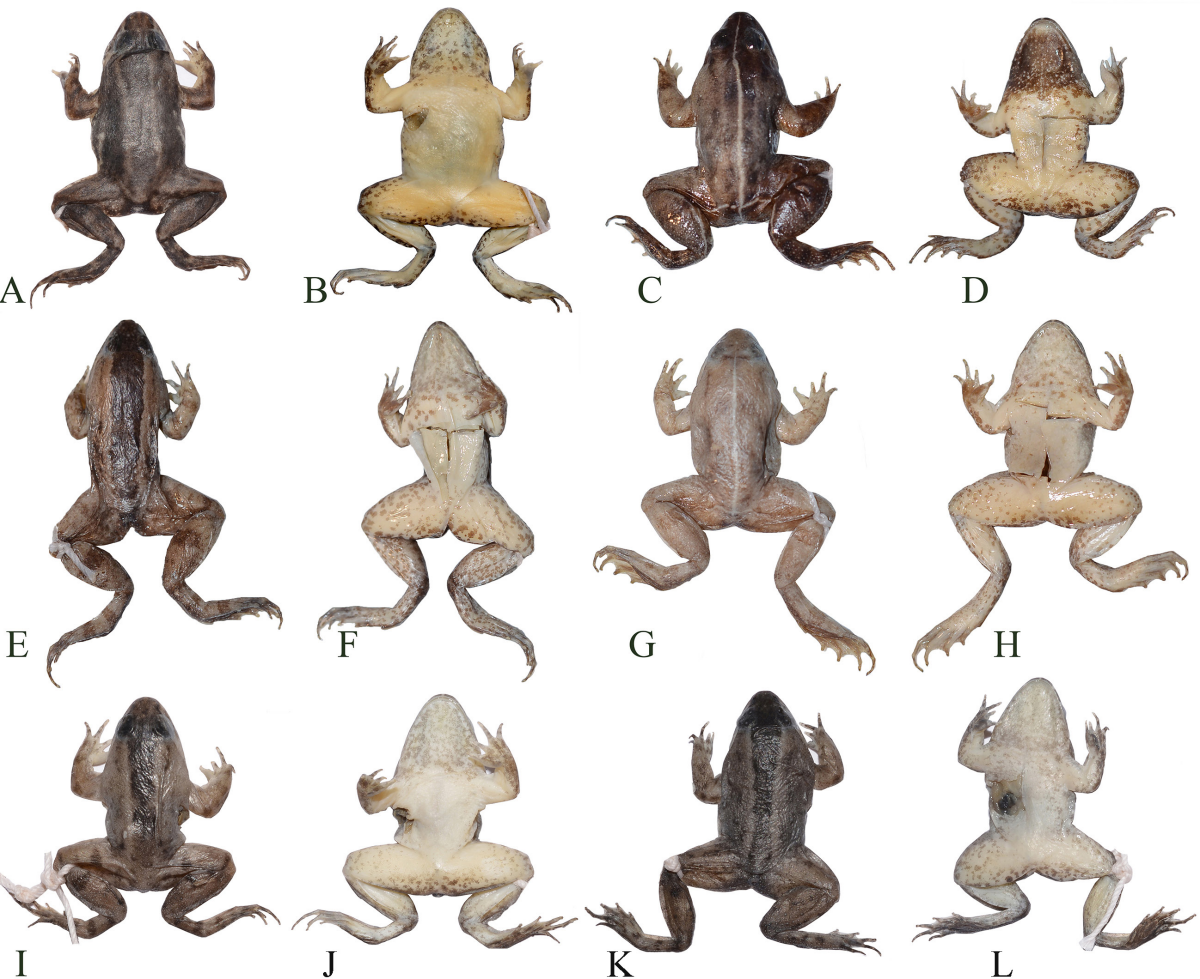
Variation of coloration in males of *Occidozyga nishikawai* sp. nov. in preservative. **A, B**. IB A.6288; **C, D**. IB A.6291; **E, F**. IB A.6289; **G, H**. IB A.6290; **I, J**. IB A. 6293; **K, L**. IB A 6292.

The measurements of the type series are given in Table 3. Females are larger than males, dorsal coloration varies in life, from pale brown to dark brown; mid-dorsal stripe present in almost all specimens but varies among individuals, distinct or indistinct (Figs 4, 5).

##### Male secondary sexual characteristics.

Vocal sac absent; in the breeding season, a single, pale yellow, swollen, and granular nuptial pad on the dorsal surface of finger I. Males slightly smaller than females (SVL 23.76–27.50 mm in males, *n* = 5 and 27.86–34.60 in females, *n* = 8) (Table 3).

##### Natural history notes.

**Figure 6.**
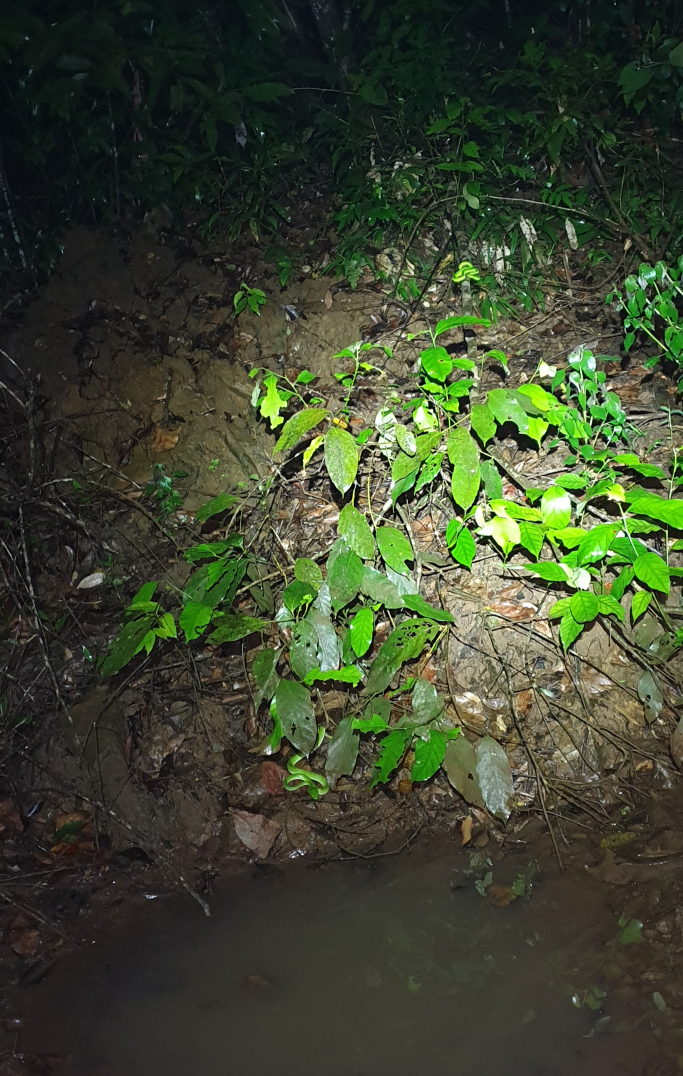
Habitat of the new *Occidozyga* species, in which the holotype was found in Kon Plong Commune, Quang Ngai Province, Central Vietnam; the vegetation also was inhabited by green pit vipers (*Trimeresurus* sp.), visible lower left in the photograph.

The holotype was discovered while it was calling from a puddle in the forest near a residential area. There were no paddy rice fields in the surroundings. The paratypes were found in an open land with grass and bushes, ~ 30 m away from a nearby rice field. Another frog species, *Microhyla cf. pulchra*, was also found around the puddle, and two individuals of green pit vipers (*Trimeresurus* sp.) (Fig. 6).

##### Distribution and conservation status.

**Figure 7.**
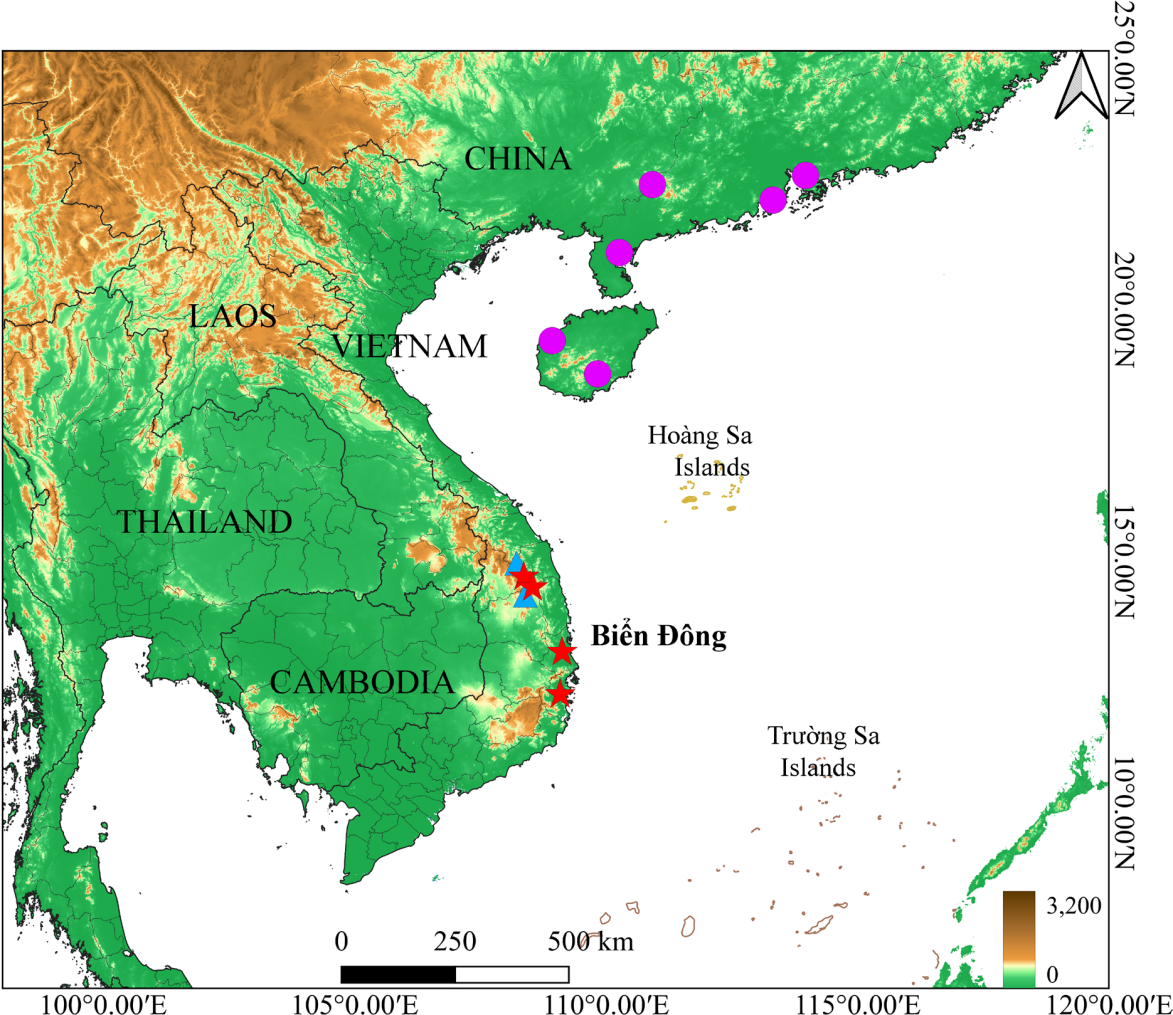
Map showing the distribution of *Occidozyga nishikawai* sp. nov. (red stars), *O. lingnanica* in the previous study (purple circles) and in this study (blue triangles).

The new species *Occidozyga nishikawai* sp. nov. is currently known from Kon Plong Commune, Quang Ngai Province; Kon Chu Rang Nature Reserve, Gia Lai Province; Hoa Thinh Commune, Dak Lak Province; and Van Ninh Commune, Khanh Hoa Province (Fig. 7). Populations of *Occidozyga* in central Vietnam were previously misidentified as *O. martensii* (Nguyen et al. 2009). Consequently, the actual distribution of *Occidozyga nishikawai* sp. nov. likely extends to neighboring provinces from where *O. martensii* was previously reported.

##### Comparisons.

We compare the new species with all species in the genus *Occidozyga* in Table 4.

**Table 4.** Comparison of morphological characters of species within the genus *Occidozyga*.

Characters	SVL (mm)	Tongue	Toes webbing	Tympanum	Tarsal fold	Tips of fingers	Tips of toes	Finger lengths	Toe lengths	Position of tibia-tarsal articulation	Cusp on mandible	Ventral coloration	Dorsal skin texture	References
*Occidozyga nishikawai* sp. nov.	Males: 23.76–27.50 Females: 27.86–34.60	Tongue rounded, without a notch	Mostly webbed	Tympanum moderately distinct	Absent	Rounded without discs	Rounded, lacking discs	II<I<IV<III	I<II<V<III<IV	Reaching at the posterior margin of supratympanic fold	Absent	Creamy white ventral surface with brown spots scattered	Smooth, with small warts on flanks, upper arm, thigh, and tibia, dorsal stripe broad, dark brown, running from tip of snout to vent, limbs with darker cross-bars	This study
***O. baluensis***	Males: 16–26 Females: 33–34	Unknown	3/4 toe webbed	Unknown	Present	Unknown	Pear-shaped toe discs	II=I	Unknown	Reaching the tip of the snout	A single tooth-like projection at the tip of the mandible	White with black largely marbled	Skin smooth, dark brown above, limbs with darker cross-bars	Boulenger 1896
***“O. berbeza”***	Males: 16–18 Females: 18–19	Tongue narrow, rounded posteriorly	Only first and second toes webbed to discs	Tympanum hidden or only anterior rim visible	Present	Rounded, lacking discs	Tips of toes with discs	II<I<IV<III	I<V<II<III<IV	Reaching, at most, posterior border of the eye	Mandible with a single median cusp	Creamy with large scattered dark gray spots on throat and posterior of thigh	Orange-brown; dorsal stripe broad, dark brown, running from tip of snout posteriorly, and diverging at sacrum, continuing across the groin to femur and tibia; dorsal skin with transverse wrinkles	Matsui et al. 2021
***O. celebensis***	Female: 33	Rounded behind	Fully webbed	Hidden	Unknown	Bluntly pointed	Small discs	Unknown	Unknown	Reaching to midway between shoulder and eye	Unknown	Yellowish	Skin smooth, with a few scattered, rounded warts above; olive above with indistinct darker markings	Smith 1927
***O. diminutiva***	Male: 18. Female: 26.4	Tongue slightly notched	Unknown	Unknown	Unknown	No discs	Absent	Unknown	Unknown	Unknown	A single tooth-like, projection at the tip of the mandible	Blotched (white and brown)	Skin of back, smooth or faintly shagreened; a thin dorsolateral skin fold from eye halfway to groin	Taylo, 1922; Matsui et al. 2021; Chan et al. 2021
***O. floresiana***	Males: 35–37 Females: 40–51	Tongue with a notch	Fully webbed	Unknown	Unknown	Terminal discs	Distinctly enlarged, flattened toe discs	II<I<IV	Unknown	Unknown	A single bony protuberance at the lower jaw tip	Unknown	Unknown	Mertens 1927; Trageser et al. 2021; Matsui et al. 2021
***O. laevis***	Males: 21–37.4 Females: 31.6–48	Rounded behind	Fourth toe broadly webbed to disk	Tympanum covered, with skin, dimly visible	Present	Discs present	Toe tips with disc	I<II	Unknown	Reaching slightly in front of the eye	Unknown	Uniform white or with few scattered punctate spots on breast	Skin on snout smooth; on occiput and back finely corrugate; above olive-brown to brown, with two darker lines following dim dorsolateral folds;	Günther 1858; Taylor 1962
***O. lima***	Males: 22–26 Females: 26–32	Tongue slender, worm-like	Fully webbed	Hidden	Present	Pointed	Toe tips with discs	Unknown	Unknown	Reaching tympanum	A single tooth-like projection at the tip of the mandible	Venter with pair of dark chin stripes	Dorsum strongly tuberculate; bold black horizontal stripe on rear of thighs	Taylor 1962
***O. lingnanica***	Males: 19.9-21.8 Females: 26.8-28.5	Tongue wide and swollen	Toes with full webs	Tympanum hidden, edge invisible	Absent	Fingers rounded, not dilated and without disks	Tips of toes rounded, dilated into rounded disks	II=I<IV<III	I < II < V < III < IV	Reaching at the posterior margin of supratympanic fold	Absent	Creamy with grey mottling in throat and with greyish speckles in belly	Rough, large tubercles scattered on dorsum and flanks, dorsal limbs smoother; a faint fold across head between eyes	This study, Lyu et al. 2022
***O. magnapustulosa***	Males: 17–20 Females: 14–21.3	Tongue oval, wider posteriorly than anteriorly	4/5 toe webbed	Tympanum moderately distinct	Present	No discs	Toes with only slight development of terminal discs	II<I	Unknown	Reaching middle of the eye	Unknown	Venter without distinct granules; below, chin and throat brownish with some lighter flecks; venter and part of underside of thighs nearly immaculate	Body with large craterlike pustules: brown above with two vague paler areas on middle of back preceded and followed by slightly, darker areas or lines	Taylor and Elbel 1958
***O. martensii***	Male: 25.5	Rounded behind without a notch	Fully webbed, but deeply indented webs	Tympanum covered with skin, but most of the outline distinct	Present	Discs present	Toes with terminal discs	II=IV<I<III	I<II<III<V<IV	Reaching the eye	Unknown	Venter yellowish-white to flesh white, but with a fine peppering of darkish pigment, somewhat denser on chin and throat, and outer parts of the underside of thighs	Skin with a few small scattered tubercles posteriorly on dorsum, nearly smooth on anterior part of dorsum and head; above brown-gray with dark spots; a dark band between the back of the eyes	Peters 1867; Taylor 1962
***O. myanhessei***	Males: 22.0–27.4 Females: 28.4–31.3	Tongue fleshy, rounded, without a notch	Fully webbed	Tympanum concealed, tympanic rim weakly elevated relative to tympanum	Present	Rounded, not expanded into discs	Rounded, slightly expanded into discs	II<IV<I<III	I<II<V<III<IV	Unknown	Unknown	Venter pale neutral gray with smoky white stipples	Skin on top of head and on dorsum, and flank smooth; dorsal and lateral ground color of head and body dark-gray with indistinct raw umber blotches and mottling	Köhler et al. 2021
***O. obscura***	Males: 24.2–27.5 Females: 31.5–32.2	Tongue narrow and slender, unnotched	Fully webbed	Tympanum hidden, edge invisible	Unkown	Pointed, not dilated	Pointed, dilated into pear-shaped disks	I<II<IV<III;	I<II<III<V<IV	Reaching between the posterior and anterior of the eye	Absent	Creamy white; gular with a pair of distinct or indistinct longitudinal dark stripes	Creamy white; gular with a pair of distinct or indistinct longitudinal dark stripes	Lyu et al. 2022
***O. shiwandashanensis***	Males: 25.2–33.8 Females: 34.9–38.9	Tongue fleshy, rounded, without a notch	Fully webbed, but the fourth toe not webbed to disk	Hidden	Present	Pointed	Rounded, slightly expanded into a disk	II<I<IV<III	I<II<V<III<IV	Reaching to posterior of the eye	Absent	Creamy white ventral surface with brown spots on lateral margin and throat	Dorsal surface shagreened with small and raised tubercles, more evident on flanks; pale brown dorsum with irregular pale dark spots, especially on head	Chen et al. 2022
***O. semipalmata***	Males: 22.8–40.9 Females: 35–48	Large and rounded behind	2/3 webbed	Hidden	Present (feeble)	Very small discs	Toe tips with discs	I<II	V<III	Reaching the anterior border of the eye	Unknown	Venter greyish-white, the throat, and limbs speckled with brown	Skin smooth, olive-brownish above, with indistinct darker markings; dorsum uniformly colored, no light dorsolateral stripe	Smith 1927
***O. sumatrana***	Males: 20–31 Females: 35–48	Unknown	Fully webbed	Unknown	Unknown	No discs	Toe tips with discs	I=II<III<IV	Unknown	Unknown	A single tooth-like projection at the tip of the mandible	Venter yellow with scattered brown spots; blackish gular	Dorsum grey-brown or olive-brown, usually mottled dark brown; dark brown band on either side of the cloaca	Peters 1877
***O. swanbornorum***	Males: 23.2–28.6 Female: 30.6	Rounded tongue without a notch	Moderately webbed	Small and indistinct tympanum	Absent	Rounded without discs	Toe discs feebly developed	IV<II<I<III	I<V<II<III<IV	Unknown	Absent	Venter uniform cream white, becoming brown with white mottling and groups of minute dark-grey flecks present in the gular region	Dorsal tubercles on flanks tipped in brownish white to white; granularly textured dorsum with scattered tubercles, dorsal tubercles on flanks tipped in brownish white to white, and the absence of a lateral line	Trageser et al. 2021
***O. tompotika***	Males: 28.3 Female: 34.1	Rounded, no median notch behind	Half webbed toes	Tympanic annulus hidden	Present	Wide expansions at finger tips	Wide expansions at toe tips	II<I<IV<III	I<II<V<III<IV	Unknown	Unknown	Ventral surface marbled with dark brown and gular, surfaces more heavily marked	Dorsal skin rough but no large tubercles; dark brown or blackish, usually without faint markings, pattern, or mottling	Iskandar et al. 2011

*Occidozyga nishikawai* sp. nov. can be distinguished from *O. baluensis* by the absence of a tarsal fold vs present; relative length of fingers I and II (II<I vs I=II); tibia–tarsal articulation reaching the posterior margin of supratympanic rim vs reaching the tip of the snout; ventral creamy white with brown spots scattered vs white with heavy black marbling.

*Occidozyga nishikawai* sp. nov. differs from *O. berbeza* by having a larger size (SVL 23.76–27.50 mm in males, 27.86–34.60 mm in females vs 16.0–18.0 mm in males, 18.0–19.0 mm in females, respectively); tarsal fold absent vs present; tibio-tarsal articulation reaching the posterior margin of supratympanic rim vs reaching, at most, posterior border of the eye; cusp on mandible absent vs a single median cusp (Matsui et al. 2021).

*Occidozyga nishikawai* sp. nov. differs from *O. celebensis* by having dorsal surface smooth vs a few scattered; toes 2/3 webbed vs fully webbed; tips of toes without discs vs small discs; dorsal surface smooth, with small warts on flanks vs a few scattered, round warts above, upper arm, thigh, and tibia; dorsal stripe broad, dark-brown, limbs with darker cross-bars vs olive with indistinct darker markings (Smith 1927).

*Occidozyga nishikawai* sp. nov. differs from *O. diminutiva* by having a larger size (SVL 23.76–27.50 mm in males, 27.86–34.60 mm in females vs 18.0 mm in males, 26.4 mm in females, respectively); tongue round posteriorly vs slightly notched posteriorly; cusp on mandible absent vs a single tooth-like projection at the tip of mandible (Taylor 1922).

*Occidozyga nishikawai* sp. nov. differs from *O. floresiana* by having a smaller size (SVL 23.76–27.50 mm in males, 27.86–34.60 mm in females vs 35.0–37.0 mm in males, 40.0–51.0 mm in females, respectively); tongue round posteriorly vs notched posteriorly; toes two-thirds webbed vs fully webbed; cusp on mandible absent vs a single bony protuberance at the lower jaw tip (Mertens 1927).

*Occidozyga nishikawai* sp. nov. differs from *O. laevis* by tarsal fold absent vs present; tips of fingers and toes without discs vs with discs; relative length of fingers I and II (II<I vs I<II) (Günther 1858).

*Occidozyga nishikawai* sp. nov. differs from *O. lima* by having toes 2/3 webbed vs fully webbed; tarsal fold absent vs present; tips of toes without discs vs with discs; cusp on mandible absent vs a single tooth-like projection at the tip of the mandible; ventral creamy white with brown spots vs venter with pair of dark chin stripes; dorsal surface smooth vs strongly tuberculate (Kuhl and Van Hasselt 1822).

*Occidozyga nishikawai* sp. nov. differs from *O. lingnanica* by having a larger size (SVL 23.76–27.50 mm in males, 27.86–34.60 mm in females vs 19.9–22.1 mm in males, 26.8–28.8 mm in females, respectively); tips of fingers and toes without discs vs with discs; relative length of fingers I and II (II<I vs I=II); dorsal surface smooth vs relatively rough (Lyu et al. 2022).

*Occidozyga nishikawai* sp. nov. differs from *O. magnapustulosa* by having a larger size (SVL 23.76–27.50 mm in males, 27.86–34.60 mm in females vs 17.0–20.0 mm in males, 14.0–21.3 mm in females, respectively); tarsal fold absent vs present; tibia-tarsal articulation reaching the posterior margin of supratympanic rim vs reaching middle of the eye; dorsum slightly smooth vs large craterlike pustules (Taylor and Elbel 1958).

*Occidozyga nishikawai* sp. nov. differs from *O. martensii* by having tongue round posteriorly vs notched posteriorly; tarsal fold absent vs present; tips of fingers without discs vs with discs; relative length of fingers and toes (II<I<IV<III in fingers, I<II<V<III<IV in toes vs II=IV<I<III in fingers, I<II<III<V<IV in toes, respectively); tibia-tarsal articulation reaching the posterior margin of supratympanic vs reaching to the eye; venter creamy white with scattered brown spots vs venter yellowish white to fresh white with a somewhat fine peppering of darkish pigment (Peters, 1867; Taylor, 1962).

*Occidozyga nishikawai* sp. nov. differs from *O. myanhessei* by having toes 2/3 webbed vs fully webbed; tarsal fold absent vs present; tips of toes without discs vs slightly expanded into discs; different relative length of fingers (II<I<IV<III vs II<IV<I<III) (Köhler et al. 2021).

*Occidozyga nishikawai* sp. nov. differs from *O. obscura* by having dorsum relatively smooth vs rough; toes two-thirds webbed vs fully webbed; difference of relative length of toes (I<II<V<III<IV vs I<II<III<V<IV) (Lyu et al. 2022).

*Occidozyga nishikawai* sp. nov. differs from *O. shiwandashanensis* by the absence of tarsal fold vs present; tibia-tarsal articulation reaching the posterior margin of supratympanic rim vs reaching to the posterior of the eye; dorsal surface slightly smooth vs shagreened (Chen et al. 2022).

*Occidozyga nishikawai* sp. nov. differs from *O. semipalmata* by the absence of tarsal fold vs feeble tarsal fold; tips of fingers without discs vs with small discs; tibia-tarsal articulation reaching the posterior of supratympanic rim vs reaching the anterior border of the eye (Smith 1927).

*Occidozyga nishikawai* sp. nov. differs from *O. sumatrana* by having toes 2/3 webbed vs fully webbed; tips of toes without discs vs with discs; relative length of fingers I and II (II<I vs I=II); cusp on mandible absent vs a single tooth-like projection at the tip of the mandible; venter creamy white with brown spots scattered vs yellow with brown spots; dorsal stripe broad, dark-brown vs dorsum grey-brown or olive-brown, usually mottled dark brown; dark brown band on either side of the cloaca (Peters 1877).

*Occidozyga nishikawai* sp. nov. differs from *O. swanbornorum* by having head longer than wide vs head wider than long (HL/HW 1.02–1.12 in males and 1.05–1.14 in females vs HL/HW 0.68–0.75 in males and 0.76 in female); tips of toes without discs vs with discs; relative length of fingers and toes (II<I<IV<III in fingers, I<II<V<III<IV in toes vs IV<II<I<III in fingers, I<V<II<III<IV in toes, respectively) (Trageser et al. 2021).

*Occidozyga nishikawai* sp. nov. differs from *O. tompotika* by having tongue round posteriorly vs notched posteriorly; dorsal surface relatively smooth vs dorsal skin rough (Iskandar et al. 2011).

##### Etymology.

We name this new species in honor of Professor Dr. Kanto Nishikawa, from Kyoto University, Japan, in recognition of his support of our herpetological research and conservation in Vietnam. As a common name for the species, we suggest Nishikawa’s puddle frog (English), and Cóc nước ni-shi-ka-wa (Vietnamese).

#### Occidozyga lingnanica

Taxon classificationAnimaliaAnuraDicroglossidae

Lyu & Wang, 2022

72F7BC81-06CA-54C0-91C8-74DA8FF97E2F

##### Specimens examined.

**Vietnam** • ♂; Kon Plong Commune, Quang Ngai Province, Vietnam; 1.180 m a.s.l.; April 2023; H. T. Ninh and N. Orlov leg.; IB A.6489 (field number KP.2023.8); • ♀; Kon Ka Kinh National Park, Gia Lai Province, Vietnam; 926 m a.s.l.; May 2023; H. T. Ninh and N.Orlov leg.; IB A.6490 (field number KKK.2023.80).

##### Body and head.

Body stocky, size small (SVL 22.31 mm in male and 25.93 mm in female). Head longer than wide (HW/HL 0.93 in male and 0.99 in female; snout round in dorsal view; canthus rostralis round, loreal region oblique; nostril round and located at the middle between tip of snout and eye (S-NL/N-EL 0.98 in male and 0.97 in female); eye orientation laterally, pupil diamond-shaped; interorbital space slightly narrower than internarial distance (IND/IOD 1.06 in male and 1.04 in female); tympanum rim indistinct; vomerine teeth absent; tongue round posteriorly (Table 2).

Forelimbs short and robust; hand length shorter than body length (HAL/SVL 0.41 in male and 0.38 in female), relatively thin and long, relative finger lengths II=I<IV<III; tips of fingers round, not dilated and without discs; fingers without webbing and fringes; subarticular tubercles present at the base of each finger, prominent and round; supernumerary tubercles absent; inner and outer palmar tubercles prominent and round.

Hind limbs robust, heels not meeting when hind limbs flexed at right angles to the axis of the body; tibio-tarsal articulation reaching at the posterior margin of supratympanic fold when hind limb is stretched along the side of the body; toes distinctly long and thin, relative toe lengths I < II < V < III < IV; tips of toes round, dilated into round disks; toes 2/3 webbed, metatarsal web present, distinct lateral fringes on toes I and V; subarticular tubercles round, prominent; inner metatarsal tubercle large and long-elliptic, distinct, length triple the width; outer metatarsal tubercle absent; tarsal fold absent.

Skin on dorsal surface rough, large tubercles scattered on dorsum and flanks, dorsal surface of limbs smoother; a faint fold across head between eyes; supratympanic fold present, extending from the posterior corner of the eye to the previous arm; pineal ocellus absent; dorsolateral fold absent; ventral surface smooth.

##### Coloration in life.

**Figure 8.**
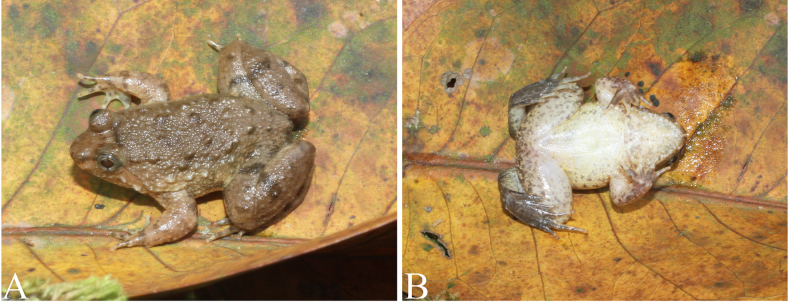
Dorsal view (**A**) and ventral view (**B**) of *Occidozyga lingnanica* (IB A.6489) in life.

Dorsal surface greyish brown with irregular black mottling; dorsal surface of limbs with dark grey transverse bars; supratympanic fold dark brown. Pupil bordered with yellowish; iris brown. Throat creamy with grey mottling; chest and belly creamy white with greyish speckles; ventral surface of limbs greyish pink with grey speckles. The nuptial pad light yellow, slightly transparent (Fig. 8).

##### Coloration in preservative.

Dorsal surface fading to greyish; and ventral side of body cream, grey speckles faded into greyish.

##### Natural history notes.

One specimen was found in a small puddle in Kon Ka Kinh National Park, Gia Lai Province and another one was collected in a wetland with many shrubs and grass near the rice paddy in Kon Plong Commune, Quang Ngai Province. Males were calling at the water’s surface.

## Discussion

The successful delimitation of *Occidozyga nishikawai* as a distinct species, in this study reinforces the effectiveness of an integrative approach combining morphology and phylogeny in assessing diversity within the genus *Occidozyga* (Chan et al. 2021; Flury et al. 2021; Matsui et al. 2021; Trageser et al. 2021; Lyu et al. 2022). With the description of *O. nishikawai* and the new country record of *O. lingnanica*, the total number of known species in the genus rises to 18, and the number of species recorded in Vietnam increases to five, namely *O. nishikawai*, *O. lingnanica*, *O. lima*, *O. martensii*, and *O. shiwandashanensis*.

In this study, we observed that *O. lingnanica* occurs syntopically with *Occidozyga nishikawai*, sharing similar microhabitats, therefore further research is needed to understand their ecological characteristics and determine potential niche segregation. In this respect, our study demonstrates that *Occidozyga* species also inhabit natural water bodies in addition to agricultural habitats, a finding that aligns with previous observations (Lyu et al. 2022).

The new country record for *O. lingnanica* for Vietnam presented in this study further shows that the species’ distribution ranges from southern China and Hainan Island to Central Vietnam and thus presumably should soon be proven from similar microhabitats in northern Vietnam as well.

## Supplementary Material

XML Treatment for Occidozyga nishikawai

XML Treatment for Occidozyga lingnanica

## Additional information

### Use of AI

No use of AI was reported.

### Author ORCIDs

Tao Thien Nguyen https://orcid.org/0000-0002-5640-4536

Cuong The Pham https://orcid.org/0000-0001-5158-4526

Chung Van Hoang https://orcid.org/0000-0002-0709-974X

Vien Hong Thi Nguyen https://orcid.org/0000-0002-3744-3054

Dang Trong Do https://orcid.org/0000-0003-3048-1434

Truong Quang Nguyen https://orcid.org/0000-0002-6601-0880

Thomas Ziegler https://orcid.org/0000-0002-4797-609X

### Data availability

All of the data that support the findings of this study are available in the main text.
